# Open Access Week, 24 to 30 October

**DOI:** 10.2807/1560-7917.ES.2016.21.43.30383

**Published:** 2016-10-27

**Authors:** Eurosurveillance editorial team

**Affiliations:** 1 European Centre for Disease Prevention and Control (ECDC), Stockholm, Sweden

Open Access Week, starting 24 October 2016, is a worldwide event established to promote the potential benefits of open access and to foster wider participation in helping to make open access the norm in research and information sharing.

In its eighth year, Open Access Week presents an opportunity for funding bodies, universities and others to use the context to advance policy changes in favour of open access on a local level, thereby adding to the global momentum.

Read more about Open Access Week here

